# Global, regional, and national burden and trends of early‐onset tracheal, bronchus, and lung cancer from 1990 to 2019

**DOI:** 10.1111/1759-7714.15227

**Published:** 2024-02-01

**Authors:** Jun Ma, Ying‐da Song, Xiao‐ming Bai

**Affiliations:** ^1^ Department of Thoracic Surgery, Shanxi Provincial People's Hospital Taiyuan China; ^2^ Fifth Clinical Medical College Shanxi Medical University Taiyuan People's Republic of China

**Keywords:** disability‐adjusted life years (DALYs), early‐onset tracheal, bronchus, and lung cancer (EO‐TBL), global burden of disease (GBD), incidence, mortality

## Abstract

**Background:**

Tracheal, bronchus, and lung cancer (TBL) is one of the main cancer health problems worldwide, but data on the burden and trends of early‐onset tracheal, bronchus, and lung cancer (EO‐TBL) are sparse. The aim of the present study was to provide the latest and the most comprehensive burden estimates of the EO‐TBL cancer from 1990 to 2019.

**Methods:**

Overall, we used data from the Global Burden of Disease (GBD) study in EO‐TBL cancer from 1990 to 2019. Evaluation metrics included incidence, mortality, and disability‐adjusted life years (DALYs). The joinpoint regression model was used to analyze the temporal trends. Decomposition analysis was employed to analyze the driving factors for EO‐TBL cancer burden alterations. Bayesian age‐period‐cohort (BAPC) analysis was used to estimate trends in the next 20 years.

**Results:**

The global age‐standardized incidence rate (ASIR), age‐standardized mortality rate (ASMR), and age‐standardized DALYs rate (ASDR) for EO‐TBL cancer decreased significantly from 3.95 (95% uncertainty interval [UI]: 3.70–4.24), 3.41 (95% UI: 3.19–3.67), 158.68 (95% UI: 148.04–170.92) in 1990 to 2.82 (95% UI: 2.54–3.09), 2.28 (95% UI: 2.07–2.49), 106.47 (95% UI: 96.83–116.51) in 2019 with average annual percent change (AAPC) of −1.14% (95% confidence interval [CI]: −1.32 to −0.95), −1.37% (95% CI: −1.55 to −1.18), and − 1.35% (95% CI: −1.54 to −1.15) separately. The high and high‐middle sociodemographic index (SDI) region had a higher burden of EO‐TBL cancer but demonstrated a downward trend. The most prominent and significant upward trends were Southeast and South Asia, Africa, and women in the low SDI and low‐middle SDI quintiles. At the regional and national level, there were significant positive correlations between ASDR, ASIR, ASMR, and SDI. Decomposition analysis showed that population growth and aging have driven the increase in the number of incidence, mortality, and DALYs in the global population, especially among the middle SDI quintile and the East Asia region. The BAPC results showed that ASDR, ASIR, and ASMR in women would increase but the male population remained relatively flat over the next 20 years.

**Conclusions:**

Although global efforts have been the most successful and effective in reducing the burden of EO‐TBL cancer over the past three decades, there was strong regional and gender heterogeneity. EO‐TBL cancer need more medical attention in the lower SDI quintiles and in the female population.

## Graphical abstract

Tracheal, bronchus, and lung cancer (TBL) is one of the main cancer health problems worldwide, but data on the burden and trends of EO‐TBL cancer are sparse. Our aim was to provide the latest and the most comprehensive burden estimates of the EO‐TBL cancer from 1990 to 2019. Although global efforts have been the most successful and effective in reducing the burden of EO‐TBL cancer over the past three decades, there is strong regional and gender heterogeneity. EO‐TBL cancer needs more medical attention in the lower SDI quintiles and in the female population.

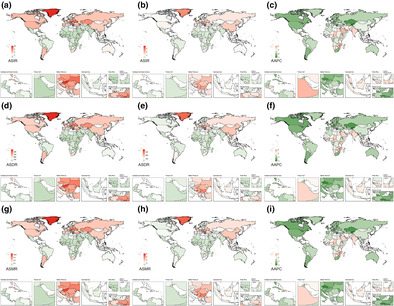

## INTRODUCTION

Lung cancer, which accounts for 13% of all cancer cases and 23% of all cancer‐related deaths worldwide, is the most common cause of cancer‐related deaths globally and represents a primary public health problem worldwide.^1^ Cancer is a disease of older adults with more than 50% of malignancies diagnosed in patients over 65 years of age,^2^, ^3^ but the early‐onset cancer incidence among people under age of 50 is increasing worldwide, showing a trend in the younger population.^4^ Fewer studies have looked at early‐onset cancer, particularly early‐onset lung cancer. Early‐onset tracheal, bronchus, and lung (EO‐TBL) cancer is defined as tracheal, bronchus, and lung cancer diagnosed before the age of 50 and it is supposed that it has a particularly strong genetic component.^5^, ^6^, ^7^ A large genome‐wide association study (GWAS) evaluated four potential risk sites for early‐onset non‐small cell lung cancer in the Chinese population.^7^ These racial differences in early‐onset lung cancer were explored in the Surveillance, Epidemiology and End Results (SEER) program;^8^, ^9^ familial aggregation and gender differences have also been observed simultaneously.^10^, ^11^, ^12^ In addition, the polymorphism of hMLH1 rs1799977, microsomal epoxide hydrolase gene and matrix metalloproteinase 1 can lead to early‐onset lung cancer.^13^, ^14^, ^15^ CYP1B1 and CYP2A13 genotypes could be related to early‐onset lung cancer in women.^12^ Although previous studies have focused on the basic medical and individual levels, to date, few studies have investigated early‐onset lung cancer at a worldwide epidemiology and disease burden level.

The Global Burden of Disease (GBD) study 2019 has provided a systematic resource for epidemiological research, including risk factor exposure and attributable burden worldwide, with stratification based on age, sex, cause, and location.^16^ The GBD study refers to lung cancer as tracheal, bronchus, and lung cancer.^17^ These data have provided a better understanding of the epidemiological trend and disease burden of EO‐TBL cancer.

The present study aimed to describe the burden of EO‐TBL cancer in 204 countries and regions from 1990 to 2019, analyze its temporal trends, and investigate its relationship with the level of sociodemographic development, decomposition analysis, and future projections until 2040. Our study offers several insights that can inform strategies for addressing lung cancer in the early‐onset population. At the same time, our results support the value of health policy and resource allocation at regional, national, and global levels.

## METHODS

### Data sources

We performed a secondary analysis of data from the GBD 2019 study, which used available data to provide an assessment of health loss across 369 diseases and injuries by age and sex in 204 countries or territories.^16^, ^18^ The burden of disease is the overall socioeconomic and health stress that leads to poor health, disability, and premature death. We extracted the estimates and 95% uncertainty interval (UI) for incidence, mortality, and disability‐adjusted life years (DALYs) as measures of the burden of TBL cancer (International Classification of Diseases (ICD)‐10 code: C56.9). In this study, the age‐group of EO‐TBL cancer was the group composed of individuals aged <50 years.^4^ As there was no individual with TBL cancer under 10 years of age, the population with TBL cancer in the age range of 10–49 years was selected for inclusion. The 10–49‐year‐old age range was divided into 5‐year age categories. We collected the aforementioned indicators of TBL cancer from both sexes in 5‐year age‐groups (10–14‐, 15–19‐, 20–24‐, 25–29‐, 30–34‐, 35–39‐, 40–44‐, and 45–49‐year‐olds) and by the five development levels regions, 21 GBD regions, and 204 countries/regions (including all member countries of the World Health Organization). The sociodemographic index (SDI) was a composite indicator of social and economic conditions for each location and year that included total fertility rate, mean education for those aged 15 years and older, and lag‐distributed income per person, and calculated the geometric mean of these three measures for each location.

### Statistical analysis

The estimates were standardized by age to the population with EO‐TBL cancer using the direct standardization method. The estimated relationships between age‐standardized DALYs rate (ASDR), age‐standardized incidence rate (ASIR), age‐standardized mortality rate (ASMR), and SDI were evaluated using Pearson's correlation analysis. We used the joinpoint regression model to analyze the temporal trends in ASDR, ASIR, and ASMR for EO‐TBL cancer from 1990 to 2019.^19^ Decomposition analysis was used to parse out the drivers of changes in EO‐TBL cancer burden from 1990 to 2019, we evaluated the relative contribution of three factors: population growth, aging, and epidemiological change.^20^ We applied a Bayesian age‐period‐cohort (BAPC) model to further predict the ASDR, ASIR, and ASMR of new EO‐TBL cancer from 2020 to 2040.^21^ ASDR, ASIR, and ASMR are reported per 100 000 population, data are reported as values with 95% UI. Temporal trends from 1990 to 2019 were assessed using Joinpoint software (version 5.0.2) from the National Cancer Institute. The statistical software R (version 4.2.2) was used for all statistical analyses and mapping. A two‐sided *p*‐value <0.05 was defined as the significance threshold.

## RESULTS

### Trend analysis

**FIGURE 1:**
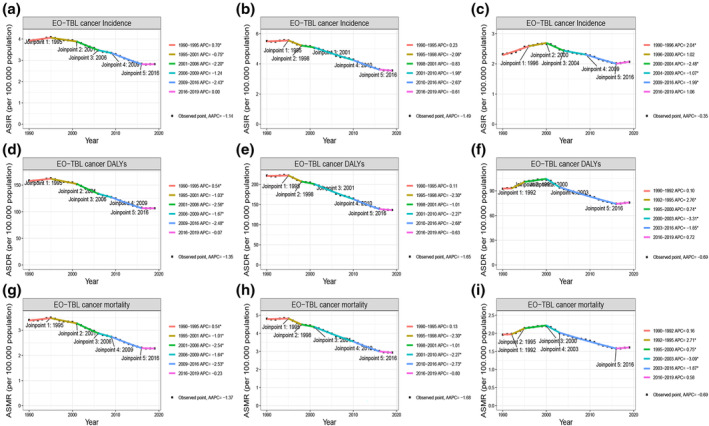
Joinpoint regression analysis of global EO‐TBL cancer ASIR in both (a), males (b), and females (c). Joinpoint regression analysis of global EO‐TBL cancer ASDR in both (d), males (e), and females (f). Joinpoint regression analysis of global EO‐TBL cancer ASMR in both (g), males (h), and females (i). *Indicates that the APC is significantly different from zero at the alpha = 0.05 level. Final selected model: five joinpoints. EO‐TBL cancer, early‐onset tracheal, bronchus, and lung cancer cancer; DALYs, disability‐adjusted life‐years; AAPC, average annual percent change; APC, annual percentage change; ASIR, age‐standardized incidence rate; ASMR, age‐standardized mortality rate; ASDR, age‐standardized DALYs rate.

From 1990 to 2019, the global ASIR, ASMR and ASDR of EO‐TBL cancer demonstrated significantly decreasing trends (ASIR: average annual percent change [AAPC] = −1.14%; 95% confidence interval [CI]: −1.32 to −0.95; ASMR: AAPC = −1.37%; 95% CI: −1.55 to −1.18; ASDR: AAPC = −1.35%; 95% CI: −1.54 to −1.15) (Table 1). The global ASIR, ASMR and ASDR of EO‐TBL cancer increased between 1990 and 1995, but continued to decline at a faster rate from 1995 to 2016 (Figure 1a,d,g).

**TABLE 1:** Incident, mortality, and DALYs of EO‐TBL cancer in 2019, and AAPC from 1990 to 2019, by sex, SDI quintile, and region level.

Location	Incidence			Mortality			DALYs		
	Incident cases (95% UI)	ASIR (95% UI)	AAPC% (95%CI), 1990–2019	Mortality cases (95% UI)	ASMR (95% UI)	AAPC% (95% CI), 1990–2019	DALYs cases (95% UI)	ASDR (95% UI)	AAPC% (95% CI), 1990–2019
Global	137227.66 (123632.78 to 150577.08)	2.82 (2.54 to 3.09)	−1.14 (−1.32 to −0.95)	110938.6979 (100858.02 to 121398.32)	2.28 (2.07 to 2.49)	−1.37 (−1.55 to −1.18)	5163978.38 (4696137.18 to 5650067.31)	106.47 (96.83 to 116.51)	−1.35 (−1.54 to −1.15)
Male	87426.28 (77089.95 to 98519.81)	3.56 (3.14 to 4.01)	−1.49 (−1.77 to −1.2)	72156.06 (64265.96 to 80910.96)	2.94 (2.62 to 3.29)	−1.68 (−1.95 to −1.4)	3343324.26 (2980286.20 to 3749090.80)	136.65 (121.82 to 153.23)	−1.65 (−1.92 to −1.39)
Female	49801.38 (43747.39 to 56096.31)	2.07 (1.82 to 2.33)	−0.35 (−0.65 to −0.05)	38782.63 (34592.04 to 43540.51)	1.61 (1.44 to 1.81)	−0.69 (−1.02 to −0.36)	1820654.12 (1623958.35 to 2042054.49)	75.86 (67.65 to 85.09)	−0.69 (−0.99 to −0.39)
High SDI	20761.05 (18505.21 to 23189.61)	3.00 (2.68 to 3.35)	−1.84 (−1.98 to −1.7)	14177.80222 (13434.02 to 14950.44)	2.04 (1.94 to 2.16)	−2.32 (−2.45 to −2.18)	644519.06 (610078.76 to 680191.87)	93.95 (88.85 to 99.25)	−2.28 (−2.44 to −2.12)
High‐middle SDI	39927.41 (35347.63 to 44882.63)	3.83 (3.39 to 4.31)	−1.41 (−1.67 to −1.16)	31395.98261 (28171.35 to 34955.80)	3.00 (2.69 to 3.34)	−1.76 (−2.24 to −1.27)	1451426.12 (1302398.49 to 1617123.47)	140.22 (125.75 to 156.36)	−1.73 (−2.19 to −1.27)
Middle SDI	51428.62 (44372.46 to 58759.80)	3.16 (2.73 to 3.61)	−0.56 (−0.79 to −0.34)	43023.46139 (37335.52 to 49146.63)	2.64 (2.30 to 3.01)	−0.82 (−1.14 to −0.5)	2009404.51 (1745524.46 to 2291308.09)	124.26 (107.99 to 141.66)	−0.83 (−1.14 to −0.51)
Low‐middle SDI	19020.16 (16625.33 to 21518.30)	1.87 (1.63 to 2.11)	0.13 (−0.02 to 0.27)	16839.13422 (14775.30 to 19060.77)	1.66 (1.46 to 1.88)	0.04 (−0.1 to 0.18)	797195.68 (698835.53 to 902837.95)	78.11 (68.50 to 88.43)	0.05 (−0.06 to 0.16)
Low SDI	6017.63 (4939.01 to 7420.36)	1.23 (1.01 to 1.51)	0.32 (0.23 to 0.4)	5440.525844 (4464.93 to 6754.93)	1.12 (0.92 to 1.39)	0.31 (0.23 to 0.39)	258534.86 (212225.36 to 320258.17)	52.08 (42.75 to 64.65)	0.33 (0.24 to 0.41)
Andean Latin America	605.25 (429.08 to 831.18)	1.60 (1.13 to 2.19)	−0.99 (−1.57 to −0.4)	517.45 (368.00 to 709.07)	1.37 (0.97 to 1.87)	−1.04 (−1.63 to −0.45)	25316.33 (17910.66 to 34858.24)	182.53 (152.08 to 217.80)	−0.99 (−1.57 to −0.4)
Australasia	515.92 (389.30 to 681.17)	2.68 (2.02 to 3.54)	−1.57 (−1.79 to −1.35)	329.20 (282.52 to 383.07)	1.70 (1.46 to 1.99)	−1.62 (−1.83 to −1.41)	14962.42 (12770.15 to 17522.07)	128.93 (103.36 to 154.74)	−1.57 (−1.79 to −1.35)
Caribbean	711.10 (556.03 to 893.42)	2.43 (1.90 to 3.05)	−1.23 (−1.49 to −0.98)	603.92 (473.48 to 760.83)	2.06 (1.62 to 2.60)	−1.27 (−1.53 to −1.01)	28107.17 (21954.13 to 35538.84)	148.76 (126.33 to 172.56)	−1.23 (−1.49 to −0.98)
Central Asia	1674.35 (1437.71 to 1962.51)	2.96 (2.54 to 3.47)	−2.63 (−3.03 to −2.24)	1457.96 (1253.92 to 1708.30)	2.58 (2.22 to 3.02)	−2.68 (−2.99 to −2.36)	69469.81 (59602.60 to 81556.74)	146.84 (91.17 to 234.96)	−2.63 (−3.03 to −2.24)
Central Europe	3822.27 (3251.68 to 4458.40)	4.63 (3.94 to 5.40)	−2.15 (−2.42 to −1.89)	3174.05 (2681.70 to 3689.39)	3.82 (3.23 to 4.45)	−2.14 (−2.41 to −1.88)	142744.08 (120701.27 to 166013.57)	122.47 (105.11 to 143.70)	−2.15 (−2.42 to −1.89)
Central Latin America	2236.06 (1830.99 to 2715.04)	1.45 (1.19 to 1.76)	−1.16 (−1.4 to −0.91)	1884.18 (1541.14 to 2298.92)	1.22 (1.00 to 1.49)	−1.18 (−1.43 to −0.93)	90241.13 (73958.65 to 109945.77)	66.58 (47.15 to 91.58)	−1.16 (−1.4 to −0.91)
Central sub‐Saharan Africa	978.72 (560.23 to 1830.76)	1.80 (1.03 to 3.36)	−0.55 (−0.66 to −0.44)	888.41 (510.27 to 1666.60)	1.65 (0.95 to 3.09)	−0.55 (−0.66 to −0.44)	41591.32 (23876.76 to 77943.61)	96.50 (75.30 to 122.09)	−0.55 (−0.66 to −0.44)
East Asia	54903.37 (45332.36 to 65634.83)	4.91 (4.06 to 5.85)	−0.85 (−1.13 to −0.58)	43371.50 (36029.62 to 51920.64)	3.85 (3.20 to 4.61)	−0.87 (−1.16 to −0.57)	2014063.18 (1676798.81 to 2405840.53)	58.40 (47.87 to 71.15)	−0.85 (−1.13 to −0.58)
Eastern Europe	5912.55 (5059.63 to 6863.16)	4.06 (3.48 to 4.72)	−1.98 (−2.74 to −1.21)	4699.34 (3987.46 to 5455.73)	3.22 (2.73 to 3.73)	−2 (−3.13 to −0.86)	215658.78 (183112.43 to 250087.13)	58.45 (48.63 to 68.32)	−1.98 (−2.74 to −1.21)
Eastern Sub‐Saharan Africa	1440.04 (1121.34 to 1851.49)	0.85 (0.66 to 1.09)	0.05 (−0.02 to 0.13)	1316.08 (1027.19 to 1686.27)	0.78 (0.61 to 1.00)	0.04 (−0.04 to 0.12)	62270.29 (48500.14 to 79790.31)	78.26 (66.55 to 91.95)	0.05 (−0.02 to 0.13)
High‐income Asia Pacific	3161.88 (2691.98 to 3710.42)	2.33 (1.98 to 2.74)	−2.14 (−2.38 to −1.91)	1748.68 (1603.36 to 1920.44)	1.27 (1.16 to 1.41)	−2.14 (−2.38 to −1.91)	80098.78 (73232.35 to 88164.73)	40.99 (31.88 to 51.23)	−2.14 (−2.38 to −1.91)
High‐income North America	7022.65 (5954.08 to 8268.78)	3.06 (2.59 to 3.60)	−2.88 (−3.09 to −2.67)	4883.90 (4622.39 to 5147.00)	2.12 (2.01 to 2.24)	−2.91 (−3.12 to −2.7)	220700.23 (208572.88 to 233095.41)	100.72 (83.96 to 121.34)	−2.88 (−3.09 to −2.67)
North Africa and Middle East	8082.43 (6887.15 to 9529.51)	2.12 (1.81 to 2.50)	−0.89 (−1.09 to −0.7)	7125.28 (6053.49 to 8399.25)	1.87 (1.59 to 2.21)	−0.9 (−1.09 to −0.71)	335421.30 (284815.17 to 395981.72)	114.05 (104.81 to 123.27)	−0.89 (−1.09 to −0.7)
Oceania	245.90 (152.84 to 392.87)	3.43 (2.14 to 5.46)	0.28 (0.15 to 0.4)	218.02 (135.68 to 348.01)	3.07 (1.92 to 4.88)	0.26 (0.14 to 0.38)	10594.77 (6552.09 to 17027.15)	100.24 (79.40 to 125.92)	0.28 (0.15 to 0.4)
South Asia	14608.40 (12185.48 to 17082.42)	1.38 (1.15 to 1.61)	0.68 (0.43 to 0.93)	13063.58 (10876.19 to 15270.82)	1.24 (1.03 to 1.45)	0.66 (0.39 to 0.92)	620811.23 (516488.60 to 725992.50)	87.53 (74.31 to 103.32)	0.68 (0.43 to 0.93)
Southeast Asia	13968.41 (11053.54 to 16923.82)	3.11 (2.46 to 3.77)	−0.12 (−0.24 to −0.01)	12300.33 (9873.24 to 14760.27)	2.73 (2.20 to 3.28)	−0.15 (−0.28 to −0.02)	577151.92 (462701.88 to 692662.55)	75.59 (43.42 to 141.79)	−0.12 (−0.24 to −0.01)
Southern Latin America	1097.60 (789.82 to 1500.30)	2.57 (1.85 to 3.52)	−2.67 (−2.8 to −2.55)	923.24 (771.49 to 1110.05)	2.16 (1.80 to 2.60)	−2.72 (−2.84 to −2.6)	42846.29 (35741.06 to 51582.86)	74.32 (68.83 to 79.90)	−2.67 (−2.8 to −2.55)
Southern sub‐Saharan Africa	1076.37 (849.68 to 1350.03)	2.41 (1.91 to 3.01)	−1.88 (−2.38 to −1.37)	968.92 (767.27 to 1215.84)	2.17 (1.73 to 2.72)	−1.85 (−2.33 to −1.37)	45169.00 (35653.96 to 56837.03)	36.15 (28.20 to 46.26)	−1.88 (−2.38 to −1.37)
Tropical Latin America	2714.37 (2511.30 to 2926.76)	1.82 (1.69 to 1.97)	−1.25 (−1.44 to −1.05)	2358.13 (2185.09 to 2531.96)	1.58 (1.46 to 1.70)	−1.29 (−1.48 to −1.09)	110612.75 (102489.09 to 118880.99)	173.90 (147.02 to 202.35)	−1.25 (−1.44 to −1.05)
Western Europe	10587.38 (8683.69 to 12661.58)	3.57 (2.93 to 4.27)	−1.87 (−2.08 to −1.66)	7436.94 (6850.19 to 8010.77)	2.50 (2.30 to 2.69)	−1.87 (−2.01 to −1.72)	335109.45 (308177.74 to 361544.72)	96.78 (91.35 to 102.31)	−1.87 (−2.08 to −1.66)
Western sub‐Saharan Africa	1862.67 (1463.02 to 2319.17)	0.96 (0.75 to 1.19)	0.37 (0.23 to 0.51)	1669.58 (1297.88 to 2087.30)	0.87 (0.67 to 1.08)	0.35 (0.21 to 0.49)	81038.16 (62864.56 to 101586.92)	59.78 (54.33 to 66.28)	0.37 (0.23 to 0.51)
Andean Latin America	137227.66 (123632.78 to 150577.08)	2.82 (2.54 to 3.09)	−1.14 (−1.32 to −0.95)	110938.6979 (100858.02 to 121398.32)	2.28 (2.07 to 2.49)	−1.37 (−1.55 to −1.18)	5163978.38 (4696137.18 to 5650067.31)	106.47 (96.83 to 116.51)	−1.35 (−1.54 to −1.15)
Australasia	87426.28 (77089.95 to 98519.81)	3.56 (3.14 to 4.01)	−1.49 (−1.77 to −1.2)	72156.06 (64265.96 to 80910.96)	2.94 (2.62 to 3.29)	−1.68 (−1.95 to −1.4)	3343324.26 (2980286.20 to 3749090.80)	136.65 (121.82 to 153.23)	−1.65 (−1.92 to −1.39)
Caribbean	49801.38 (43747.39 to 56096.31)	2.07 (1.82 to 2.33)	−0.35 (−0.65 to −0.05)	38782.63 (34592.04 to 43540.51)	1.61 (1.44 to 1.81)	−0.69 (−1.02 to −0.36)	1820654.12 (1623958.35 to 2042054.49)	75.86 (67.65 to 85.09)	−0.69 (−0.99 to −0.39)
Central Asia	20761.05 (18505.21 to 23189.61)	3.00 (2.68 to 3.35)	−1.84 (−1.98 to −1.7)	14177.80222 (13434.02 to 14950.44)	2.04 (1.94 to 2.16)	−2.32 (−2.45 to −2.18)	644519.06 (610078.76 to 680191.87)	93.95 (88.85 to 99.25)	−2.28 (−2.44 to −2.12)
Central Europe	39927.41 (35347.63 to 44882.63)	3.83 (3.39 to 4.31)	−1.41 (−1.67 to −1.16)	31395.98261 (28171.35 to 34955.80)	3.00 (2.69 to 3.34)	−1.76 (−2.24 to −1.27)	1451426.12 (1302398.49 to 1617123.47)	140.22 (125.75 to 156.36)	−1.73 (−2.19 to −1.27)
Central Latin America	51428.62 (44372.46 to 58759.80)	3.16 (2.73 to 3.61)	−0.56 (−0.79 to −0.34)	43023.46139 (37335.52 to 49146.63)	2.64 (2.30 to 3.01)	−0.82 (−1.14 to −0.5)	2009404.51 (1745524.46 to 2291308.09)	124.26 (107.99 to 141.66)	−0.83 (−1.14 to −0.51)
Central Sub‐Saharan Africa	19020.16 (16625.33 to 21518.30)	1.87 (1.63 to 2.11)	0.13 (−0.02 to 0.27)	16839.13422 (14775.30 to 19060.77)	1.66 (1.46 to 1.88)	0.04 (−0.1 to 0.18)	797195.68 (698835.53 to 902837.95)	78.11 (68.50 to 88.43)	0.05 (−0.06 to 0.16)
East Asia	6017.63 (4939.01 to 7420.36)	1.23 (1.01 to 1.51)	0.32 (0.23 to 0.4)	5440.525844 (4464.93 to 6754.93)	1.12 (0.92 to 1.39)	0.31 (0.23 to 0.39)	258534.86 (212225.36 to 320258.17)	52.08 (42.75 to 64.65)	0.33 (0.24 to 0.41)
Eastern Europe	605.25 (429.08 to 831.18)	1.60 (1.13 to 2.19)	−0.99 (−1.57 to −0.4)	517.45 (368.00 to 709.07)	1.37 (0.97 to 1.87)	−1.04 (−1.63 to −0.45)	25316.33 (17910.66 to 34858.24)	182.53 (152.08 to 217.80)	−0.99 (−1.57 to −0.4)
Eastern sub‐Saharan Africa	515.92 (389.30 to 681.17)	2.68 (2.02 to 3.54)	−1.57 (−1.79 to −1.35)	329.20 (282.52 to 383.07)	1.70 (1.46 to 1.99)	−1.62 (−1.83 to −1.41)	14962.42 (12770.15 to 17522.07)	128.93 (103.36 to 154.74)	−1.57 (−1.79 to −1.35)
High‐income Asia Pacific	711.10 (556.03 to 893.42)	2.43 (1.90 to 3.05)	−1.23 (−1.49 to −0.98)	603.92 (473.48 to 760.83)	2.06 (1.62 to 2.60)	−1.27 (−1.53 to −1.01)	28107.17 (21954.13 to 35538.84)	148.76 (126.33 to 172.56)	−1.23 (−1.49 to −0.98)

*Note*: Rates are reported per 100 000 person‐years.

Abbreviations: AAPC, average annual percent change; ASIR, age‐standardized incidence rate; ASMR, age‐standardized mortality rate; ASDR, age‐standardized DALYs rate; CI, confidence interval; DALYs, disability‐adjusted life‐years; EO‐TBL cancer, early‐onset tracheal, bronchus, and lung cancer cancer; SDI, sociodemographic index; UI, uncertainty interval.

The global burden of EO‐TBL cancer measured in incidence was 137,228 cases (95% UI, 123,633–150,577) in 2019, an increase of 28.7% from 1990 (106,592, 95% UI: 99,834 to 114,531). Contrarily, the ASIR decreased from 3.95 per 100,000 population (95% UI: 3.70–4.24) in 1990 to 2.81 per 100,000 population (95% UI: 2.54–3.09) in 2019, with an AAPC of −1.14% (95% CI, −1.32 to −0.95) in the same period (Table 1). With sex‐specific classification, men suffered a heavier burden in EO‐TBL cancer, even after adjustment for age adjusted. The number of incidence cases has more than nearly doubled in men (87,426: 95% UI: 77,090–98,519) compared to women (49,801: 95% UI: 43,747–560,967) in 2019 (Table 1). However, the ASIR reduced significantly from 1990 to 2019, with a greater reduction in men than in women (males: AAPC = −1.49%; 95% CI: −1.77 to −1.2; females: AAPC = −0.35%; 95% CI: −0.65 to −0.05) (Figure 1b,c). From 1990 to 2019, worldwide mortality associated with EO‐TBL cancer increased from 91, 794 (95% UI: 85, 640–98, 849) to 110, 939 (95% UI: 100, 858–121, 398). However, ASMR continued to decline during the same period (AAPC = −1.37%; 95% CI: −1.55 to −1.18) (Table 1 and Figure 1d). The trends in the number of global mortalities and ASMR were similar to that of incidence with respect to gender (Table 1 and Figure 1e,f). Worldwide, the number of DALYs caused by EO‐TBL cancer increased from 4, 325, 261 (95% UI: 4, 029, 957–4, 664, 191) in 1990 to 5, 163, 978 (95% UI: 4, 696, 137–5, 650, 067) in 2019. The ASDR was 158.7 (95% UI: 148.04–170.92) /100, 000 population in 1990 and 106.47 (95% UI: 96.83–116.50) /100, 000 population in 2019 and decreased over the past 30 years (AAPC = −1.35%; 95% CI: −1.54 to −1.15) (Table 1 and Figure 1g). Additionally, the number of DALYs in males was significantly higher than in females and all exhibited the same trends (males: AAPC = −1.65%; 95% CI: −1.92 to −1.39; females: AAPC = −0.69%; 95% CI: −0.99 to −0.39) (Figure 1h,i).

### EO‐TBL cancer burden by spatial distribution

The region with the middle SDI quintiles had the highest incidence cases (51, 428; 95% UI: 44, 373–58, 760) and the highest mortality cases (43, 023 95% UI: 37, 336–49, 146) and the highest DALYs cases (2,009,405; 95% UI: 1, 745, 525–2, 291, 308); high‐middle SDI quintiles region had the highest ASDR, ASIR, and ASMR (ASIR = 3.82; 95% CI: 3.39–4.31; ASMR = 3.00; 95% CI: 2.69–3.34; ASDR = 140.22; 95% CI: 125.75–156.36) (Table 1). The EO‐TBL cancer burden was heavier in regions with high and high‐middle SDI, either the EO‐TBL cancer cases or rates (Table 1 and Figure S1). The EO‐TBL cancer burden by SDI quintiles region was generally lower for women than for men, but the downward trend in ASDR, ASIR, and ASMR was significantly greater in males than females (Table 2). For example, the high SDI, high‐middle SDI, and middle SDI groups of ASMR among males significantly decreased, with AAPC of −2.99%, −2.33%, and −0.92%, respectively; in contrast, females exhibited a relatively slight decline, with AAPC of −1.21%, −0.45%, and −0.69%, respectively. However, there was an upward trend in the ASDR, ASIR, and ASMR of EO‐TBL cancer from 1990 to 2019 in the low‐ and low‐middle SDI groups in females; for example, the AAPC of ASMR among females were 1.02% and 0.59% in low‐SDI and low‐middle‐SDI groups.

**TABLE 2:** AAPC in ASIR, ASDR, and ASMR of EO‐TBL cancer by sex, and SDI quintile.

		Incidence		Mortality		DALYs	
		AAPC% (95% CI)	*p*‐value	AAPC% (95% CI)	*p*‐value	AAPC% (95% CI)	*p*‐value
Male	High SDI	−2.58 (−2.76 to −2.39)	<0.001	−2.99 (−3.15 to −2.83)	<0.001	−2.96 (−3.12 to −2.8)	<0.001
	High‐middle SDI	−1.99 (−2.42 to −1.56)	<0.001	−2.33 (−2.85 to −1.82)	<0.001	−2.29 (−2.6 to −1.97)	<0.001
	Middle SDI	−0.68 (−0.94 to −0.42)	<0.001	−0.92 (−1.17 to −0.67)	<0.001	−0.87 (−1.06 to −0.68)	<0.001
	Low‐middle SDI	−0.11 (−0.29 to 0.08)	0.252	−0.15 (−0.34 to 0.04)	0.122	−0.14 (−0.34 to 0.05)	0.154
	Low SDI	0.05 (−0.03 to 0.13)	0.219	0.05 (−0.02 to 0.12)	0.2	0.07 (−0.01 to 0.14)	0.109
Female	High SDI	−0.7 (−0.82 to −0.57)	<0.001	−1.21 (−1.36 to −1.06)	<0.001	−1.19 (−1.36 to −1.03)	<0.001
	High‐middle SDI	0.09 (−0.29 to 0.46)	0.649	−0.45 (−0.85 to −0.05)	0.028	−0.46 (−0.88 to −0.04)	0.032
	Middle SDI	−0.3 (−0.64 to 0.04)	0.08	−0.69 (−1.12 to −0.26)	0.002	−0.7 (−1.09 to −0.3)	0.001
	Low‐middle SDI	0.68 (0.43–0.93)	<0.001	0.59 (0.36–0.82)	<0.001	0.57 (0.32–0.81)	<0.001
	Low SDI	1.05 (0.9–1.19)	<0.001	1.02 (0.9–1.15)	<0.001	1.02 (0.9–1.15)	<0.001

Abbreviations: AAPC, average annual percent change; ASDR, age‐standardized DALYs rate; ASIR, age‐standardized incidence rate; ASMR, age‐standardized mortality rate; CI, confidence interval; DALYs, disability‐adjusted life‐years; EO‐TBL cancer, early‐onset tracheal, bronchus, and lung cancer cancer; SDI, sociodemographic index.

At the regional level, the most significant growth in ASDR, ASIR, and ASMR occurred in South Asia, Western sub‐Saharan Africa, and Oceania. In contrast, Central Asia, high‐income North America, and Southern Latin America had the fastest decline (Table 1). Although the overall burden of EO‐TBL cancer decreased substantially from 1990 to 2019, the spatial distribution pattern remained stable (Figure S2). At the national level, in 2019, China had the highest incidence cases (53,223; 95% UI: 45,718–64,075), the most mortality cases (41,979; 95% UI: 34,620–50,649) and the most cases of DALYs (1,949,943; 95% UI: 1,612,181–2,348,116) in the world; Monaco had the most ASIR (11.6; 95% UI: 8.00–16.61), the most ASMR (8.06; 95% UI: 5.65–11.43), and the most ASDR (373.39; 95% UI: 259.63–533.41) in the world (Table S1). The most notable increases in ASDR, ASIR, and ASMR were in Lesotho and Mozambique, the most striking decline were in Kyrgyzstan and Czechia. For example, the increase of ASIR was most prominent in Lesotho (AAPC = 2.08%; 95% CI: −1.72 to −2.45), and Mozambique (AAPC = 1.87%; 95% CI: 1.70–2.03); the decrease is most pronounced in Kyrgyzstan (AAPC = −4.2%; 95% CI: −5.07 to −3.33), and Czechia (AAPC = −3.89%; 95% CI: −4.66 to −3.12) (Table S1).

**FIGURE 2:**
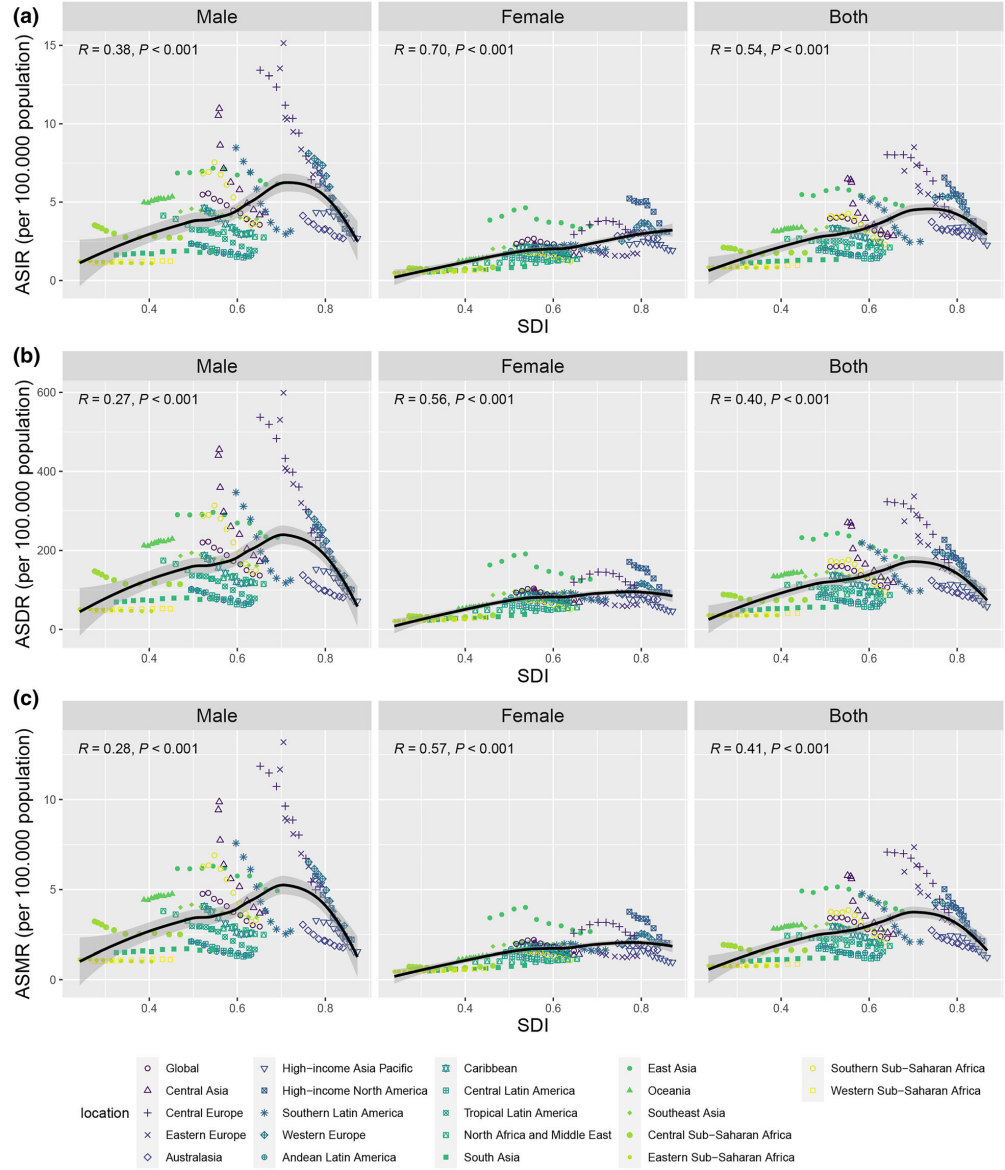
The association between EO‐TBL cancer ASIR (a, b, c), ASDR (d, e, f), and ASMR (g, h, i) and SDI at the region level for different sexes from 1990 to 2019. EO‐TBL cancer, early‐onset tracheal, bronchus, and lung cancer cancer; DALYs, disability‐adjusted life‐years; SDI, sociodemographic index; ASIR, age‐standardized incidence rate; ASMR, age‐standardized mortality rate; ASDR, age‐standardized DALYs rate.

**FIGURE 3:**
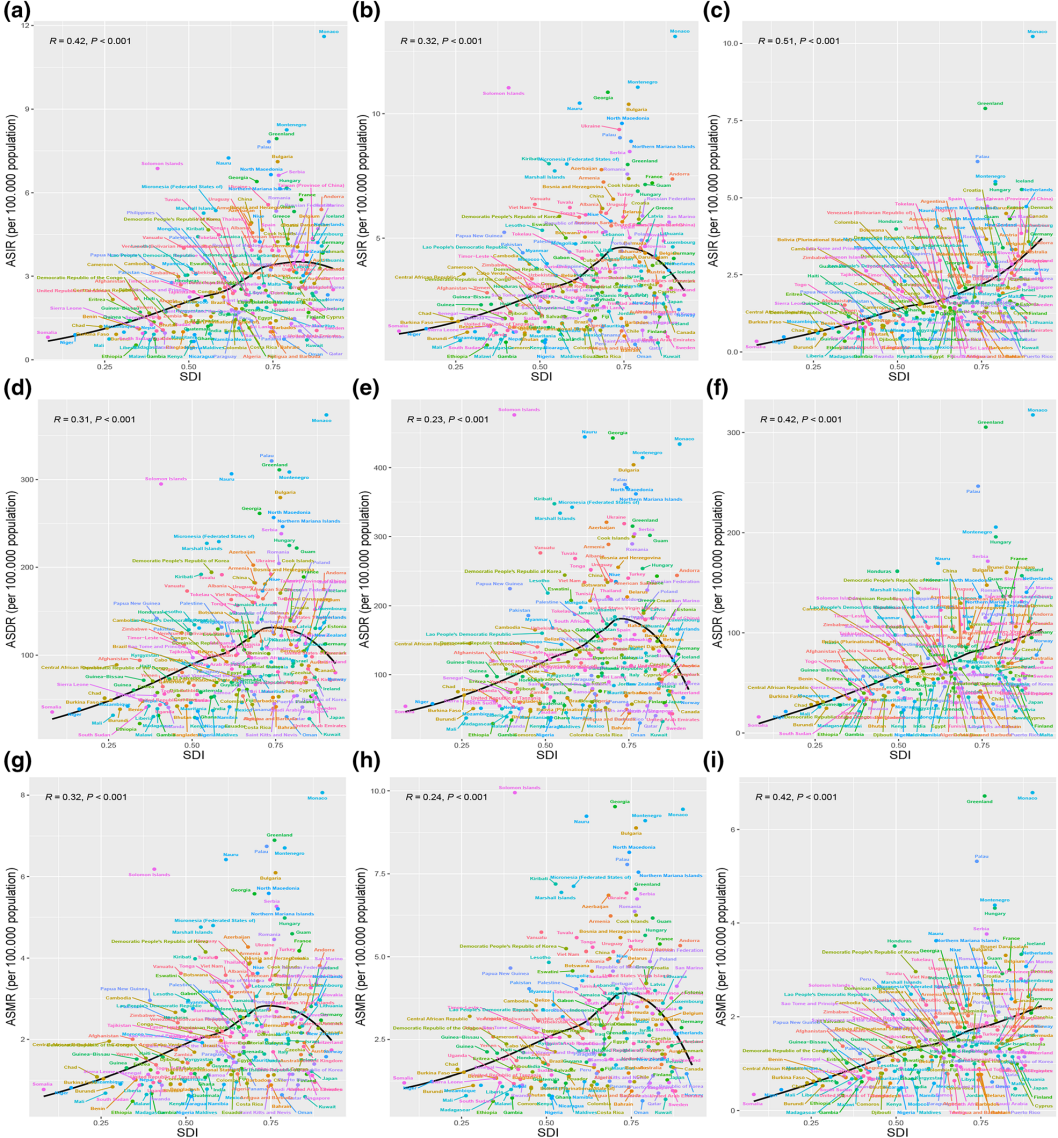
Association between ASIR (a, b, c), ASDR (d, e, f), and ASMR (g, h, i) EO‐TBL cancer and SDI at 204 countries and territories for different sexes from 1990 to 2019. EO‐TBL cancer, early‐onset tracheal, bronchus, and lung cancer cancer; DALYs, disability‐adjusted life‐years; SDI, sociodemographic index; ASIR, age‐standardized incidence rate; ASMR, age‐standardized mortality rate; ASDR, age‐standardized DALYs rate.

In the male population and the overall population, ASDR, ASIR, and ASMR increased with SDI, but then, late in the SDI level, it decreased substantially; In the female population, ASDR, ASIR, and ASMR increased as SDI increased (Figure 2). Simultaneously, these relationships were similar at regional or national level. We found significant positive correlations of SDI with ASDR, ASIR, and ASMR of EO‐TBL cancer at national level (*R* = 0.42, 0.31, and 0.32 for ASIR, ASMR, and ASDR) (Figure 3a,d,g). Moreover, AAPC of ASDR, ASIR, and ASMR generally decreased with increasing SDI (Figure S3).

### Decomposition analysis

**FIGURE 4:**
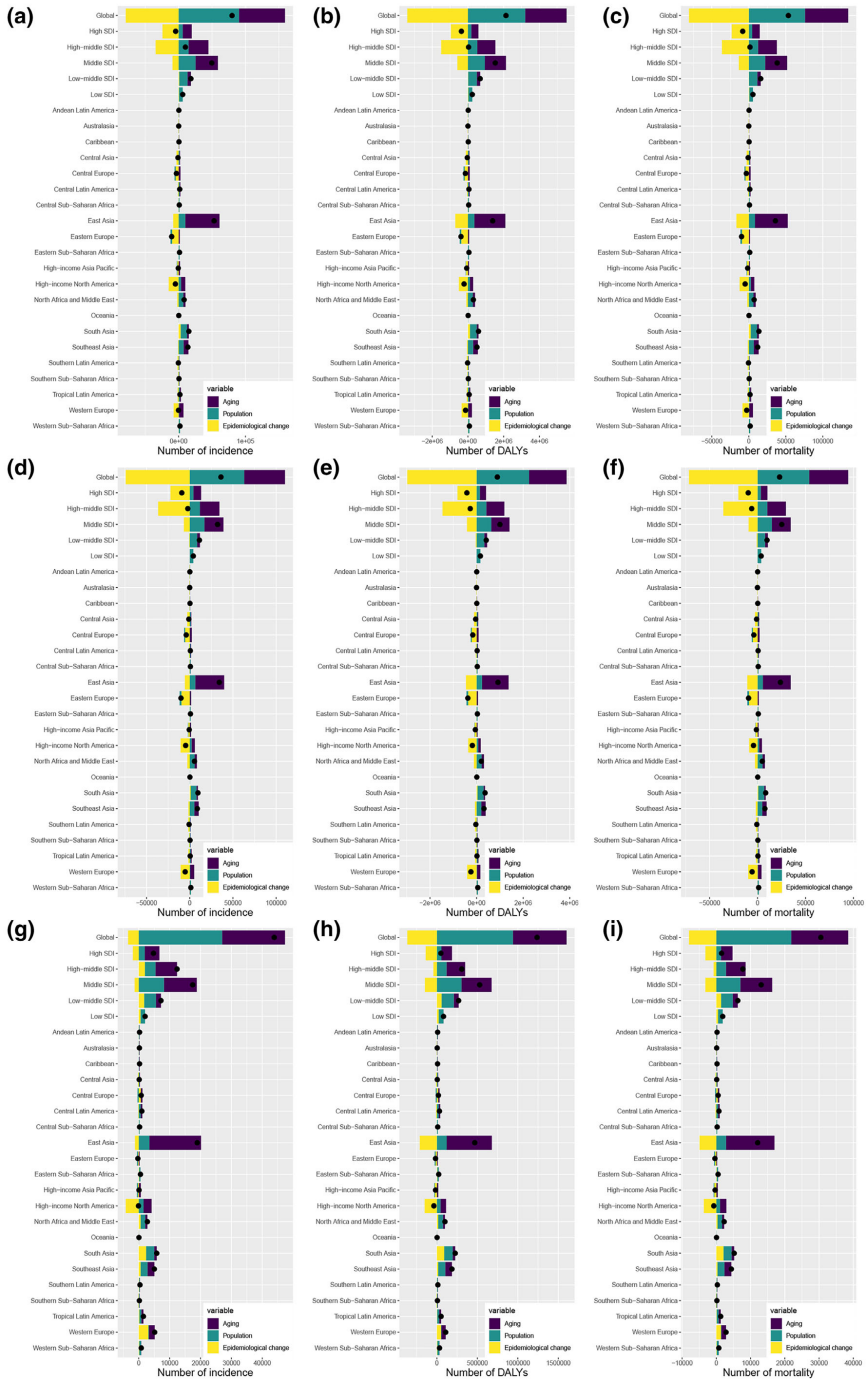
Changes in EO‐TBL cancer incidence in both (a), males (d), and females (g), DALYs in both (b), males (e), and females (h), and mortality in both (c), males (f), and females (i) according to the determinants at the population level of population growth, aging, and epidemiological change from 1990 to 2019 at the global level, SDI quintile level, and regional level. The black dots denote the sum of contribution to the changes in all three components. For each component, the magnitude of a positive value indicated a positive contribution to EO‐TBL cancer incidence, DALYs, and mortality; the magnitude of a negative value indicates a negative contribution to EO‐TBL cancer incidence, DALYs, and mortality. EO‐TBL cancer, early‐onset tracheal, bronchus, and lung cancer cancer; DALYs, disability‐adjusted life‐years; SDI, sociodemographic index.

EO‐TBL cancer has seen a steady global increase in incidence, mortality, and DALYs over the past 30 years (Figure 4). In terms of global incidence, 112.82% of the increase in EO‐TBL cancer was attributed to population growth, followed by population aging (86.17%), while epidemiological changes reduced 98.98% (Figure 4a and Table S2). Like the global incidence, this similar attribution also existed among the mortality and DALYs (Figure 4d,g). In the male population, the increased magnitude of population aging and growth was smaller than in the female population; on the other hand, the reduced magnitude of epidemiological changes in male population was greater than in female population, exhibiting a noticeable difference in sex (Figure 4b,c,e,f,h,i). At the regional level, the most significant change in EO‐TBL cancer incidence, mortality, and DALYs from 1990 to 2019 occurred in East Asia; among the five SDI regions, the most substantial changes in EO‐TBL cancer incidence, mortality, and DALYs were in the middle SDI region, while the smallest change occurred in the low SDI region (Figure 4a,d,g).

### Predictions of EO‐TBL cancer burden to 2040

**FIGURE 5:**
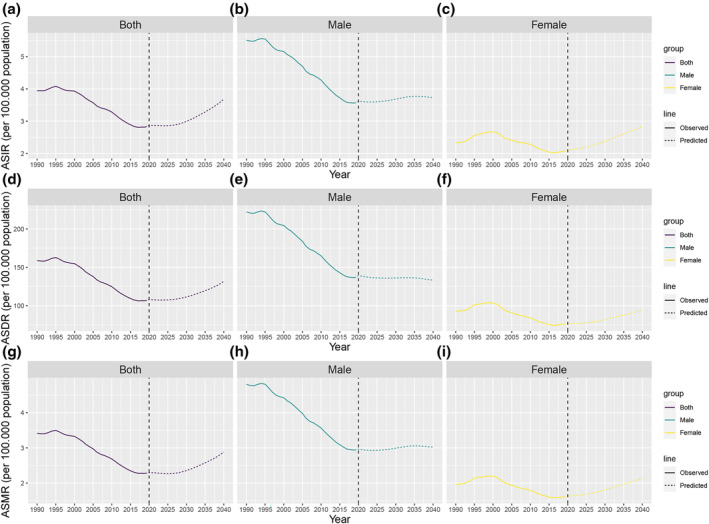
Trends in EO‐TBL cancer ASIR (a, b, c), ASDR (d, e, f), and ASMR (g, h, i) from 2019 to 2040 in different sexes predicted by BAPC models. EO‐TBL cancer, early‐onset tracheal, bronchus, and lung cancer cancer; DALYs, disability‐adjusted life‐years; BAPC, Bayesian age‐period‐cohort; ASIR, age‐standardized incidence rate; ASMR, age‐standardized mortality rate; ASDR, age‐standardized DALYs rate.

To learn about the ASIR, ASMR, and ASDR trends of EO‐TBL cancer after 2019, we used BAPC models to predict the trends Over the following two decades by sex. The ASIR, ASMR and ASDR of EO‐TBL cancer in the overall population generally follow an upward trend (Figure 5a,d,g and Table S3). The ASIR, ASMR, and ASDR of EO‐TBL cancer in women would increase annually to 2.84/100,000, 2.14/100,000, 94.44/100,000 in 2040, and there will be 80,783, 60,959, 2,656,107 cases in 2040, respectively (Figure 5c,f,i and Table S3). However, the predictive results showed that ASIR, ASMR, and ASDR of EO‐TBL cancer in men would remain fundamentally flat, from 3.56/100,000, 2.94/100,000, 136.65/100, 000 in 2019 to 3.73/100,000, 3.02/100,000, 133.26/100,000 in 2040, respectively (Figure 5b,e,h and Table S3).

## DISCUSSION

This study provides the most comprehensive and detailed assessment of the global burden of EO‐TBL cancer from 1990 to 2019. To the best of our knowledge, it is the first study to quantify the EO‐TBL cancer burden using incidence, mortality, and DALYs, which fills a gap in this field and provides new insights. Our study revealed a stable reduction in global ASDR, ASIR, and ASMR of the EO‐TBL cancer burden, while the incidence, mortality, and DALYs cases has increased over the past three decades. In the decomposition analysis, we determined that the growing EO‐TBL cancer burden is globally driven by population growth and aging. It should be noted that EO‐TBL cancer is estimated to increase worldwide over the next two decades. These findings could inform future policy interventions across age groups and regions.

We found that global new EO‐TBL cancer in ASDR, ASIR, and ASMR generally decreased in 2019 compared to 30 years ago. This report is almost the same as the previous report by the GBD 2019 respiratory tract cancer collaborators that analyzed the GBD data on respiratory tract cancers in all ages from 1990 to 2019.^17^ For the ASDR, ASIR, and ASMR of EO‐TBL cancer globally from 1990 to 2019, the results of the joinpoint regression showed that significant downward trends outweigh significant upward trends. This trend was mainly influenced by smoking patterns. With the publication of high‐quality epidemiological studies and basic research, the causal connections between tobacco and lung cancer began to be valued in the 1960s.^22^, ^23^ Smoking rates began to decline markedly in the USA and other countries with a higher human development index, a trend that translated decades later into declines in the incidence of lung cancer.^24^ Despite the fact that these trends are declining globally, from the view of ASDR, ASIR, and ASMR, the trend of the reduced EO‐TBL cancer burden masks huge country heterogeneity and the development status of different regions.

In the overall and male populations, the ASDR, ASIR, and ASMR generally increased with increasing SDI, but after the peak, showing a more drastic downward trend. Surprisingly, negative correlations were observed between AAPC and SDI. Three hypotheses have been proposed to explain these relationships. First, in many lower SDI countries or regions, due to medical technologies lagging behind, some lung cancer cases will not be detected^25^; low‐ and middle‐income countries do not consider lung cancer to be a public health priority, compared to other public health issues such as infectious diseases and accidental injuries.^26^ At the same time, weaker health systems, more limited treatment options, and lack of medical infrastructure can lead to an increased burden of EO‐TBL cancer.^17^ Second, places with rapid economic growth tend to be in middle SDI countries or regions (e.g., China, India, and Brazil), often accompanied by high rates of cigarette smoking, increasing western lifestyle and diet, and increased environmental pollution.^27^, ^28^, ^29^, ^30^, ^31^ This in turn leads to an increased burden of EO‐TBL cancer. Third, the higher SDI quintile owned more refined early screening programs, newer lung cancer therapies, healthier eating habits and more robust health care systems, consequently, can considerably reduce the burden of EO‐TBL cancer.^32^, ^33^, ^34^, ^35^

Particularly troubling were the trends among women. Although females had less burden of EO‐TBL cancer than males, there was a significantly increasing trend in females, particularly the low SDI and low‐middle SDI regions. Additionally, indicators (ASDR, ASIR, and ASMR) and SDI always showed positive correlations in women. We offer the following possible explanations for this. One is that second‐hand smoke exposure is a potential risk factor for lung cancer among never‐smokers, particularly women.^36^ Another possibility is that the carcinogen caused by solid fuels with open fires and inefficient stoves is associated with an increased risk of lung cancer, with higher contact rates among women.^37^, ^38^, ^39^ Finally, the prevalence of *EGFR* mutations was significantly higher in women compared to men, up to 43.7%.^40^, ^41^ In addition, studies have shown that higher estrogen levels can make women more likely to develop lung cancer. As a result of changes in social environment and dietary structure, increasing numbers of women have an early age at menarche and late menopause, which make the estrogen level of women at relatively high levels.^40^, ^42^

The numbers and age‐standardized rates for EO‐TBL cancer incidence, deaths, and DALYs increased substantially among adults aged 45–50 years. The guidelines of the National Comprehensive Cancer Network (NCCN) recommend the screening by lung cancer screening for individuals at high risk for lung cancer after the age of 50, but the Chinese Medical Association Lung Cancer Clinical Practice Guidelines recommend that high‐risk lung cancer populations start the screening at the age of 45.^43^ Considering that the magnitude of the increase becomes larger after age 45, the recommended age for screening can be moderately reduced while avoiding over‐diagnosis.

The trends of EO‐TBL cancer in the next two decades predicted by the BAPC models in this study indicated that the ASIR, ASMR, and ASDR in women would increase from 2019, while remaining flat in men. A recent predictive study based on Nordpred age‐period‐cohort models showed that the burden of lung cancer in 40 countries worldwide increased dramatically in women; however, the burden of lung cancer in men would continue to decline by 2035.^44^ In China, a similar trend was also found in the prediction of lung cancer incidence and mortality.^45^ Therefore, we recommend more attention to the female group.

There were a number of strengths and several limitations to this study. Reliability and accuracy of data sources which resulted in the first limitation, particularly in underdeveloped countries. Another limitation was that the GBD database did not differentiate between bronchial and lung cancers, and histological subtypes in lung cancer. The main strength of the study was that we first made a systematic estimation of the EO‐TBL cancer burden from 1990 to 2019 worldwide, according to age groups and sex, countries, regions, and SDI quintile. In addition, the joinpoint model provided more accurate temporal trends information.

In conclusion, this study provided a comprehensive estimate of the global EO‐TBL cancer burden. ASIR, ASMR, and ASDR have shown a decreasing trend between 1990 and 2019 at the global level. Over the past three decades, substantial efforts have significantly reduced the burden of EO‐TBL cancer among higher SDI countries or regions with higher SDI. However, the lower SDI quintiles and female population showed an upward trend, and women will subsequently have a higher burden of EO‐TBL cancer within the next 20 years, which should call our attention. These findings could have important implications in routine clinical practice in the future.

## AUTHOR CONTRIBUTIONS

Jun Ma participated in conceptualization, literature search, study design, data curation, data analysis, data interpretation, and drafted the original manuscript. Ying‐da Song and Jun Ma conceived of the study, and participated in its design, coordination, data collection and analysis. Jun Ma and Xiao‐ming Bai participated in study design, data curation and provided the critical revision. All authors contributed to the article and approved the submitted version.

## CONFLICT OF INTEREST STATEMENT

The authors declare that the research was conducted in the absence of commercial or financial relationships that could be construed as a potential conflict of interest.

## Supporting information


**Table S1.** Incident, mortality, and DALYs of EO‐TBL cancer in 1990 and 2019, and AAPC from 1990 and 2019 by national level. Rates are reported per 100 000 person‐years. EO‐TBL cancer, early‐onset tracheal, bronchus, and lung cancer cancer; DALYs, disability‐adjusted life‐years; ASIR, age‐standardized incidence rate; ASMR, age‐standardized mortality rate; ASDR, age‐standardized DALYs rate; UI, uncertainty interval; CI, confidence interval.

**Table S2.** Changes in incident, mortality, and DALYs number according to population‐level determinants and causes from 1990 to 2019. (a) Change in incident, mortality, or DALYs number between year 2019 and 1990. (b) Change in incident, mortality, or DALYs number due to change in the age structure. (c) Change in incident, mortality, or DALYs due to change in population number. (d) Change in incident, mortality, and DALYs due to epidemiologic changes. Epidemiologic changes refer to the incident, mortality, and DALYs number change when age structure and population hold constant. EO‐TBL cancer, early‐onset tracheal, bronchus, and lung cancer cancer; DALYs, disability‐adjusted life‐years.

**Table S3.** Prediction in ASIR, ASDR, and ASMR according to BAPC models from 2019 to 2040. EO‐TBL cancer, early‐onset tracheal, bronchus, and lung cancer cancer; DALYs, disability‐adjusted life‐years; ASIR, age‐standardized incidence rate; ASMR, age‐standardized mortality rate; ASDR, age‐standardized DALYs rate; UI, uncertainty interval.

**Figure S1.** The ASIR (A, B, C), ASDR (D, E, F), and ASMR (G, H, I) due to EO‐TBL cancer grouped by SDI quintiles for different sexes from 1990 to 2019. EO‐TBL cancer, early‐onset tracheal, bronchus, and lung cancer cancer; DALYs, disability‐adjusted life‐years; SDI, socio‐demographic index; ASIR, age‐standardized incidence rate; ASMR, age‐standardized mortality rate; ASDR, age‐standardized DALYs rate.

**Figure S2.** Global maps of ASIR (A), ASDR (D), and ASMR (G) in 1990, global maps of ASIR (B), ASDR (E), and ASMR (H) in 2019, as well as AAPC in ASIR (C), ASDR (F), and ASMR (I) from 1990 to 2019. EO‐TBL cancer, early‐onset tracheal, bronchus, and lung cancer cancer; DALYs, disability‐adjusted life‐years; AAPC, average annual percent change; ASIR, age‐standardized incidence rate; ASMR, age‐standardized mortality rate; ASDR, age‐standardized DALYs rate.

**Figure S3.** AAPC of ASIR (A), ASDR (B), and ASMR (C) from 1990 to 2019 in 204 countries and territories according to the SDI in 2019. EO‐TBL cancer, early‐onset tracheal, bronchus, and lung cancer cancer; DALYs, disability‐adjusted life‐years; AAPC, average annual percent change; SDI, socio‐demographic index; ASIR, age‐standardized incidence rate; ASMR, age‐standardized mortality rate; ASDR, age‐standardized DALYs rate.

## Data Availability

In this study, publicly available data sets were analyzed. The name and accession number can be found below: https://ghdx.healthdata.org/gbd-2019.
