# Supplementary material for: Global, regional, and national burden and trends of early‐onset tracheal, bronchus, and lung cancer from 1990 to 2019
Source: Thorac Cancer. 2024 Feb 1;15(8):601–13. doi: 10.1111/1759-7714.15227 (PMC10928250; doi:10.1111/1759-7714.15227)
Supplement: Supplementary file 2 — Table S2. Changes in incident, mortality, and DALYs number according to population‐level determinants and causes from 1990 to 2019. (a) Change in incident, mortality, or DALYs number between year 2019 and 1990. (b) Change in incident, mortality, or DALYs number due to change in the age structure. (c) Change in incident, mortality, or DALYs due to change in population number. (d) Change in incident, mortality, and DALYs due to epidemiologic changes. Epidemiologic changes refer to the incident, mortality, and DALYs number change when age structure and population hold constant. EO‐TBL cancer, early‐onset tracheal, bronchus, and lung cancer cancer; DALYs, disability‐adjusted life‐years. [file TCA-15-601-s003.docx]

**Supplemental table 2. Changes in Incidence number according to population-level determinants and causes from 1990 to 2019.**

| Location | Overll difference ^a^ | Change due to Population-level determinants (% contribute to the total changes) | | |
| --- | --- | --- | --- | --- |
|  |  | Aging ^b^ | Population ^c^ | Epidemiological change ^d^ |
| Global | 79979.52 | 68915.85 (86.17%) | 90230.07 (112.82%) | -79166.39 (-98.98%) |
| **Sex** |  |  |  |  |
| Female | 43881.65 | 20370.65 (46.42%) | 26989.29 (61.5%) | -3478.29 (-7.93%) |
| Male | 36097.88 | 47297.9 (131.03%) | 62921.04 (174.31%) | -74121.06 (-205.33%) |
| **SDI** |  |  |  |  |
| High SDI | -4433.65 | 13621.23 (-307.22%) | 6029.9 (-136%) | -24084.79 (543.23%) |
| High-middle SDI | 10159.46 | 29819.58 (293.52%) | 14759.49 (145.28%) | -34419.6 (-338.79%) |
| Middle SDI | 49645.75 | 33540.74 (67.56%) | 25288.95 (50.94%) | -9183.94 (-18.5%) |
| Low-middle SDI | 18392.7 | 5165.41 (28.08%) | 12424.38 (67.55%) | 802.91 (4.37%) |
| Low SDI | 6154.99 | -16.82 (-0.27%) | 5637.48 (91.59%) | 534.33 (8.68%) |
| **Region** |  |  |  |  |
| High-income Asia Pacific | -526.92 | 2155.31 (-409.04%) | -855.57 (162.37%) | -1826.66 (346.67%) |
| High-income North America | -4848.8 | 6345.02 (-130.86%) | 3649.6 (-75.27%) | -14843.41 (306.13%) |
| Western Europe | -154.02 | 6694.44 (-4346.36%) | 607.02 (-394.11%) | -7455.48 (4840.46%) |
| Australasia | 148.48 | 290.33 (195.54%) | 275.14 (185.31%) | -417 (-280.85%) |
| Andean Latin America | 394.29 | 227.71 (57.75%) | 414.04 (105.01%) | -247.46 (-62.76%) |
| Tropical Latin America | 2014.76 | 1802.08 (89.44%) | 1719.89 (85.36%) | -1507.21 (-74.81%) |
| Central Latin America | 1684.31 | 1240.34 (73.64%) | 1427.41 (84.75%) | -983.44 (-58.39%) |
| Southern Latin America | -399.55 | 453.77 (-113.57%) | 790.87 (-197.94%) | -1644.2 (411.51%) |
| Caribbean | 539.88 | 433.24 (80.25%) | 353 (65.38%) | -246.36 (-45.63%) |
| Central Europe | -3297.64 | 2841.08 (-86.15%) | -1787.39 (54.2%) | -4351.33 (131.95%) |
| Eastern Europe | -10423.75 | 1811.08 (-17.37%) | -2599.41 (24.94%) | -9635.41 (92.44%) |
| Central Asia | -951.24 | 858.65 (-90.27%) | 1344.74 (-141.37%) | -3154.62 (331.63%) |
| North Africa and Middle East | 8243.87 | 3397.68 (41.21%) | 6894.99 (83.64%) | -2048.8 (-24.85%) |
| South Asia | 15310.21 | 2847.31 (18.6%) | 9387.84 (61.32%) | 3075.06 (20.09%) |
| Southeast Asia | 13838.02 | 7320.23 (52.9%) | 7368.01 (53.24%) | -850.22 (-6.14%) |
| East Asia | 53117.7 | 51295.62 (96.57%) | 9908.39 (18.65%) | -8086.3 (-15.22%) |
| Oceania | 257.05 | 45.64 (17.76%) | 188.05 (73.16%) | 23.36 (9.09%) |
| Western Sub-Saharan Africa | 2080.1 | -32.53 (-1.56%) | 1961.55 (94.3%) | 151.08 (7.26%) |
| Eastern Sub-Saharan Africa | 1378.94 | 47.41 (3.44%) | 1346.64 (97.66%) | -15.11 (-1.1%) |
| Central Sub-Saharan Africa | 1017.49 | 51.56 (5.07%) | 1178.52 (115.83%) | -212.59 (-20.89%) |
| Southern Sub-Saharan Africa | 556.34 | 513.25 (92.25%) | 819.34 (147.27%) | -776.25 (-139.53%) |

**Changes in** **Mortality number according to population-level determinants and causes from 1990 to 2019.**

| Location | Overll difference ^a^ | Change due to Population-level determinants (% contribute to the total changes) | | |
| --- | --- | --- | --- | --- |
|  |  | Aging ^b^ | Population ^c^ | Epidemiological change ^d^ |
| Global | 53474.23 | 58478.03 (109.36%) | 75925.45 (141.99%) | -80929.25 (-151.34%) |
| **Sex** |  |  |  |  |
| Female | 30509.71 | 16633.46 (54.52%) | 21874.71 (71.7%) | -7998.47 (-26.22%) |
| Male | 22964.53 | 40730.42 (177.36%) | 53765.74 (234.13%) | -71531.63 (-311.49%) |
| **SDI** |  |  |  |  |
| High SDI | -8246.63 | 10287.4 (-124.75%) | 4520.41 (-54.82%) | -23054.44 (279.56%) |
| High-middle SDI | 1599.35 | 25475.27 (1592.85%) | 12456.03 (778.82%) | -36331.95 (-2271.67%) |
| Middle SDI | 38330.34 | 29760.9 (77.64%) | 22088.81 (57.63%) | -13519.36 (-35.27%) |
| Low-middle SDI | 16131.24 | 4747.88 (29.43%) | 11225.39 (69.59%) | 157.97 (0.98%) |
| Low SDI | 5611.04 | -15.62 (-0.28%) | 5175.63 (92.24%) | 451.04 (8.04%) |
| **Region** |  |  |  |  |
| High-income Asia Pacific | -1581.66 | 1469.69 (-92.92%) | -570.9 (36.09%) | -2480.46 (156.83%) |
| High-income North America | -4969.14 | 4745.82 (-95.51%) | 2715.76 (-54.65%) | -12430.72 (250.16%) |
| Western Europe | -2850.16 | 5186.26 (-181.96%) | 466.89 (-16.38%) | -8503.31 (298.34%) |
| Australasia | 2.03 | 211.15 (10398.77%) | 196.25 (9664.93%) | -405.36 (-19963.71%) |
| Andean Latin America | 325.09 | 209.21 (64.35%) | 366.99 (112.89%) | -251.11 (-77.24%) |
| Tropical Latin America | 1672.65 | 1636.75 (97.85%) | 1537.49 (91.92%) | -1501.6 (-89.77%) |
| Central Latin America | 1339.67 | 1112.22 (83.02%) | 1248.97 (93.23%) | -1021.53 (-76.25%) |
| Southern Latin America | -477.33 | 405.03 (-84.85%) | 699.51 (-146.55%) | -1581.88 (331.4%) |
| Caribbean | 420.65 | 379.88 (90.31%) | 306.47 (72.86%) | -265.7 (-63.16%) |
| Central Europe | -3370.81 | 2490.1 (-73.87%) | -1554.14 (46.11%) | -4306.77 (127.77%) |
| Eastern Europe | -9793.46 | 1540.82 (-15.73%) | -2192.2 (22.38%) | -9142.07 (93.35%) |
| Central Asia | -895.44 | 785.07 (-87.67%) | 1200.84 (-134.1%) | -2881.35 (321.78%) |
| North Africa and Middle East | 7210.38 | 3129.19 (43.4%) | 6242.47 (86.58%) | -2161.28 (-29.97%) |
| South Asia | 13681.19 | 2630.96 (19.23%) | 8533.2 (62.37%) | 2517.03 (18.4%) |
| Southeast Asia | 12029.08 | 6735.59 (55.99%) | 6651.56 (55.3%) | -1358.07 (-11.29%) |
| East Asia | 35903.44 | 44256.93 (123.27%) | 8385 (23.35%) | -16738.48 (-46.62%) |
| Oceania | 231.56 | 42.48 (18.35%) | 169.02 (72.99%) | 20.05 (8.66%) |
| Western Sub-Saharan Africa | 1887.96 | -29.99 (-1.59%) | 1791.24 (94.88%) | 126.71 (6.71%) |
| Eastern Sub-Saharan Africa | 1281 | 44.23 (3.45%) | 1243.87 (97.1%) | -7.11 (-0.55%) |
| Central Sub-Saharan Africa | 935.65 | 48.77 (5.21%) | 1088.44 (116.33%) | -201.56 (-21.54%) |
| Southern Sub-Saharan Africa | 491.89 | 471.14 (95.78%) | 746.51 (151.76%) | -725.77 (-147.55%) |

**Changes in DALYs number according to population-level determinants and causes from 1990 to 2019.**

| Location | Overll difference ^a^ | Change due to Population-level determinants (% contribute to the total changes) | | |
| --- | --- | --- | --- | --- |
|  |  | Aging ^b^ | Population ^c^ | Epidemiological change ^d^ |
| Global | 2127618.16 | 2319969.74 (109.04%) | 3201687.54 (150.48%) | -3394039.11 (-159.52%) |
| **Sex** |  |  |  |  |
| Female | 1237682.67 | 663377.57 (53.6%) | 939131.88 (75.88%) | -364826.79 (-29.48%) |
| Male | 889935.5 | 1612511.33 (181.19%) | 2250941.23 (252.93%) | -2973517.06 (-334.13%) |
| **SDI** |  |  |  |  |
| High SDI | -367211.26 | 399786.5 (-108.87%) | 186176.56 (-50.7%) | -953174.32 (259.57%) |
| High-middle SDI | 35371.06 | 1014904.86 (2869.31%) | 520310.88 (1471.01%) | -1499844.68 (-4240.32%) |
| Middle SDI | 1529756.16 | 1182641.69 (77.31%) | 944295.79 (61.73%) | -597181.32 (-39.04%) |
| Low-middle SDI | 683592.4 | 192421.51 (28.15%) | 483136.86 (70.68%) | 8034.03 (1.18%) |
| Low SDI | 244094.25 | 285.72 (0.12%) | 222598.33 (91.19%) | 21210.2 (8.69%) |
| **Region** |  |  |  |  |
| High-income Asia Pacific | -70631.53 | 58193.75 (-82.39%) | -23953.52 (33.91%) | -104871.76 (148.48%) |
| High-income North America | -218431.81 | 179476.06 (-82.17%) | 111119.26 (-50.87%) | -509027.13 (233.04%) |
| Western Europe | -133798.1 | 202259.11 (-151.17%) | 19130.57 (-14.3%) | -355187.78 (265.47%) |
| Australasia | -40.26 | 8136.57 (-20211.75%) | 8072.29 (-20052.08%) | -16249.12 (40363.83%) |
| Andean Latin America | 14258.19 | 8458.23 (59.32%) | 16376.65 (114.86%) | -10576.69 (-74.18%) |
| Tropical Latin America | 67953.47 | 65340.26 (96.15%) | 65220.22 (95.98%) | -62607.01 (-92.13%) |
| Central Latin America | 55264.63 | 44457.21 (80.44%) | 54521.64 (98.66%) | -43714.23 (-79.1%) |
| Southern Latin America | -20751.68 | 16272.83 (-78.42%) | 29291.12 (-141.15%) | -66315.63 (319.57%) |
| Caribbean | 16343.64 | 14959.75 (91.53%) | 12934.9 (79.14%) | -11551.01 (-70.68%) |
| Central Europe | -145990.72 | 100123.26 (-68.58%) | -63784.44 (43.69%) | -182329.54 (124.89%) |
| Eastern Europe | -391563.45 | 69353.65 (-17.71%) | -90068.01 (23%) | -370849.08 (94.71%) |
| Central Asia | -36359.17 | 32867.26 (-90.4%) | 50963.96 (-140.17%) | -120190.38 (330.56%) |
| North Africa and Middle East | 303571.1 | 128729.82 (42.41%) | 267056.1 (87.97%) | -92214.83 (-30.38%) |
| South Asia | 590504.19 | 107071.34 (18.13%) | 368890.64 (62.47%) | 114542.21 (19.4%) |
| Southeast Asia | 503366.41 | 270488.22 (53.74%) | 283956.93 (56.41%) | -51078.73 (-10.15%) |
| East Asia | 1385963.1 | 1739415.61 (125.5%) | 356209.4 (25.7%) | -709661.91 (-51.2%) |
| Oceania | 10155.39 | 1720.39 (16.94%) | 7487.67 (73.73%) | 947.32 (9.33%) |
| Western Sub-Saharan Africa | 82730.8 | -1091.68 (-1.32%) | 77592.07 (93.79%) | 6230.42 (7.53%) |
| Eastern Sub-Saharan Africa | 55886.81 | 2180.06 (3.9%) | 53676.17 (96.04%) | 30.58 (0.05%) |
| Central Sub-Saharan Africa | 39724.49 | 2143.4 (5.4%) | 46068.05 (115.97%) | -8486.97 (-21.36%) |
| Southern Sub-Saharan Africa | 19462.67 | 19581.6 (100.61%) | 31840.83 (163.6%) | -31959.77 (-164.21%) |

a.Change in incident, mortality, or DALYs number between year 2019 and 1990

b.Change in incident, mortality, or DALYs number due to change in the age structure

c.Change in incident, mortality, or DALYs due to change in population number

d.Change in incident, mortality, and DALYs due to epidemiologic changes. Epidemiologic changes refer to the incident, mortality, and DALYs number change when age structure and population hold constant.

Abbreviations: EO-TBL cancer, early-onset tracheal, bronchus, and lung cancer cancer; DALYs, disability-adjusted life-years.
