# Supplementary material for: Global, regional, and national burden and trends of early‐onset tracheal, bronchus, and lung cancer from 1990 to 2019
Source: Thorac Cancer. 2024 Feb 1;15(8):601–13. doi: 10.1111/1759-7714.15227 (PMC10928250; doi:10.1111/1759-7714.15227)
Supplement: Supplementary file 3 — Table S3. Prediction in ASIR, ASDR, and ASMR according to BAPC models from 2019 to 2040. EO‐TBL cancer, early‐onset tracheal, bronchus, and lung cancer cancer; DALYs, disability‐adjusted life‐years; ASIR, age‐standardized incidence rate; ASMR, age‐standardized mortality rate; ASDR, age‐standardized DALYs rate; UI, uncertainty interval. [file TCA-15-601-s005.docx]

**Supplemental table 3. Prediction in ASIR, ASDR, and ASMR according to BAPC models from 2019 to 2040.**

|  | **incidence cases** | **ASIR** | **Mortality cases** | **ASMR** | **DALYs cases** | **ASDR** |
| --- | --- | --- | --- | --- | --- | --- |
| **Both** | | | | | | |
| **2020** | 141938.02 | 2.87 | 113915.14 | 2.30 | 5329313.43 | 108.05 |
| **2021** | 143126.24 | 2.87 | 114656.06 | 2.29 | 5355953.93 | 107.74 |
| **2022** | 144572.56 | 2.87 | 115629.95 | 2.29 | 5389396.33 | 107.47 |
| **2023** | 146392.21 | 2.86 | 116936.91 | 2.28 | 5433928.45 | 107.34 |
| **2024** | 148534.37 | 2.86 | 118524.83 | 2.27 | 5488487.84 | 107.37 |
| **2025** | 150961.31 | 2.86 | 120344.63 | 2.27 | 5551767.05 | 107.56 |
| **2026** | 153657.75 | 2.87 | 122378.06 | 2.27 | 5622467.74 | 107.90 |
| **2027** | 156681.83 | 2.89 | 124667.92 | 2.28 | 5702110.15 | 108.45 |
| **2028** | 160191.94 | 2.91 | 127339.26 | 2.30 | 5796350.73 | 109.25 |
| **2029** | 164144.50 | 2.95 | 130352.22 | 2.32 | 5904190.54 | 110.27 |
| **2030** | 168268.97 | 2.99 | 133487.94 | 2.36 | 6016270.00 | 111.46 |
| **2031** | 172340.35 | 3.04 | 136571.63 | 2.39 | 6124933.05 | 112.82 |
| **2032** | 176107.48 | 3.10 | 139403.58 | 2.43 | 6222867.80 | 114.34 |
| **2033** | 179414.27 | 3.16 | 141862.47 | 2.48 | 6306114.49 | 116.00 |
| **2034** | 182242.24 | 3.22 | 143937.08 | 2.53 | 6374696.79 | 117.77 |
| **2035** | 184534.85 | 3.29 | 145594.81 | 2.57 | 6426637.05 | 119.59 |
| **2036** | 186352.46 | 3.35 | 146895.05 | 2.63 | 6463870.49 | 121.49 |
| **2037** | 187806.06 | 3.42 | 147932.57 | 2.68 | 6491598.64 | 123.56 |
| **2038** | 188841.56 | 3.50 | 148667.64 | 2.74 | 6510380.23 | 125.91 |
| **2039** | 189513.88 | 3.59 | 149138.62 | 2.81 | 6522884.55 | 128.63 |
| **2040** | 190119.31 | 3.68 | 149572.74 | 2.88 | 6537799.88 | 131.78 |
| **Male** | | | | | | |
| **2020** | 90238.49 | 3.62 | 73871.53 | 2.96 | 3459984.41 | 139.15 |
| **2021** | 90617.64 | 3.61 | 74062.90 | 2.95 | 3466528.01 | 138.36 |
| **2022** | 91171.02 | 3.60 | 74419.97 | 2.94 | 3478027.61 | 137.61 |
| **2023** | 91976.53 | 3.60 | 75014.45 | 2.93 | 3497223.24 | 137.00 |
| **2024** | 92981.82 | 3.60 | 75794.38 | 2.93 | 3522719.97 | 136.57 |
| **2025** | 94142.58 | 3.60 | 76708.09 | 2.93 | 3552974.63 | 136.24 |
| **2026** | 95432.80 | 3.61 | 77729.65 | 2.94 | 3586347.93 | 135.96 |
| **2027** | 96905.35 | 3.62 | 78900.41 | 2.95 | 3624201.90 | 135.80 |
| **2028** | 98669.78 | 3.64 | 80309.06 | 2.96 | 3670615.35 | 135.82 |
| **2029** | 100691.33 | 3.66 | 81922.70 | 2.97 | 3724931.57 | 135.95 |
| **2030** | 102792.03 | 3.68 | 83593.72 | 2.99 | 3780977.41 | 136.14 |
| **2031** | 104825.38 | 3.71 | 85206.80 | 3.01 | 3833572.58 | 136.34 |
| **2032** | 106631.00 | 3.73 | 86630.27 | 3.03 | 3877863.20 | 136.52 |
| **2033** | 108102.46 | 3.75 | 87777.51 | 3.04 | 3911221.25 | 136.65 |
| **2034** | 109218.46 | 3.77 | 88630.86 | 3.05 | 3933667.18 | 136.63 |
| **2035** | 109940.39 | 3.77 | 89165.78 | 3.06 | 3943819.58 | 136.38 |
| **2036** | 110315.42 | 3.77 | 89429.25 | 3.06 | 3943048.77 | 135.89 |
| **2037** | 110423.74 | 3.77 | 89491.12 | 3.05 | 3934899.46 | 135.26 |
| **2038** | 110249.90 | 3.76 | 89340.40 | 3.04 | 3920245.15 | 134.58 |
| **2039** | 109829.43 | 3.74 | 89002.25 | 3.03 | 3900804.18 | 133.90 |
| **2040** | 109336.19 | 3.73 | 88613.90 | 3.02 | 3881692.78 | 133.26 |
| **Female** | | | | | | |
| **2020** | 51699.53 | 2.11 | 40043.61 | 1.63 | 1869329.02 | 76.38 |
| **2021** | 52508.61 | 2.12 | 40593.16 | 1.64 | 1889425.92 | 76.53 |
| **2022** | 53401.54 | 2.14 | 41209.97 | 1.65 | 1911368.72 | 76.69 |
| **2023** | 54415.67 | 2.15 | 41922.46 | 1.66 | 1936705.20 | 76.90 |
| **2024** | 55552.55 | 2.17 | 42730.45 | 1.67 | 1965767.88 | 77.21 |
| **2025** | 56818.73 | 2.20 | 43636.54 | 1.69 | 1998792.42 | 77.62 |
| **2026** | 58224.96 | 2.23 | 44648.41 | 1.70 | 2036119.82 | 78.17 |
| **2027** | 59776.48 | 2.26 | 45767.51 | 1.73 | 2077908.25 | 78.87 |
| **2028** | 61522.16 | 2.29 | 47030.21 | 1.75 | 2125735.38 | 79.73 |
| **2029** | 63453.18 | 2.33 | 48429.52 | 1.78 | 2179258.97 | 80.72 |
| **2030** | 65476.94 | 2.38 | 49894.22 | 1.81 | 2235292.59 | 81.79 |
| **2031** | 67514.97 | 2.42 | 51364.83 | 1.84 | 2291360.47 | 82.94 |
| **2032** | 69476.48 | 2.47 | 52773.30 | 1.87 | 2345004.60 | 84.15 |
| **2033** | 71311.80 | 2.51 | 54084.96 | 1.90 | 2394893.24 | 85.40 |
| **2034** | 73023.78 | 2.56 | 55306.22 | 1.94 | 2441029.61 | 86.67 |
| **2035** | 74594.46 | 2.61 | 56429.03 | 1.97 | 2482817.48 | 87.90 |
| **2036** | 76037.05 | 2.65 | 57465.79 | 2.00 | 2520821.72 | 89.10 |
| **2037** | 77382.32 | 2.70 | 58441.45 | 2.03 | 2556699.19 | 90.31 |
| **2038** | 78591.66 | 2.74 | 59327.24 | 2.07 | 2590135.08 | 91.58 |
| **2039** | 79684.46 | 2.79 | 60136.37 | 2.10 | 2622080.38 | 92.95 |
| **2040** | 80783.12 | 2.84 | 60958.84 | 2.14 | 2656107.10 | 94.44 |

Abbreviations: EO-TBL cancer, early-onset tracheal, bronchus, and lung cancer cancer; DALYs, disability-adjusted life-years; ASIR, age-standardized incidence rate; ASMR, age-standardized mortality rate; ASDR, age-standardized DALYs rate; BAPC, Bayesian age-period-cohort.
