# Supplementary figures and images for: Global, regional, and national burden and trends of early‐onset tracheal, bronchus, and lung cancer from 1990 to 2019
Source: Thorac Cancer. 2024 Feb 1;15(8):601–13. doi: 10.1111/1759-7714.15227 (PMC10928250; doi:10.1111/1759-7714.15227)

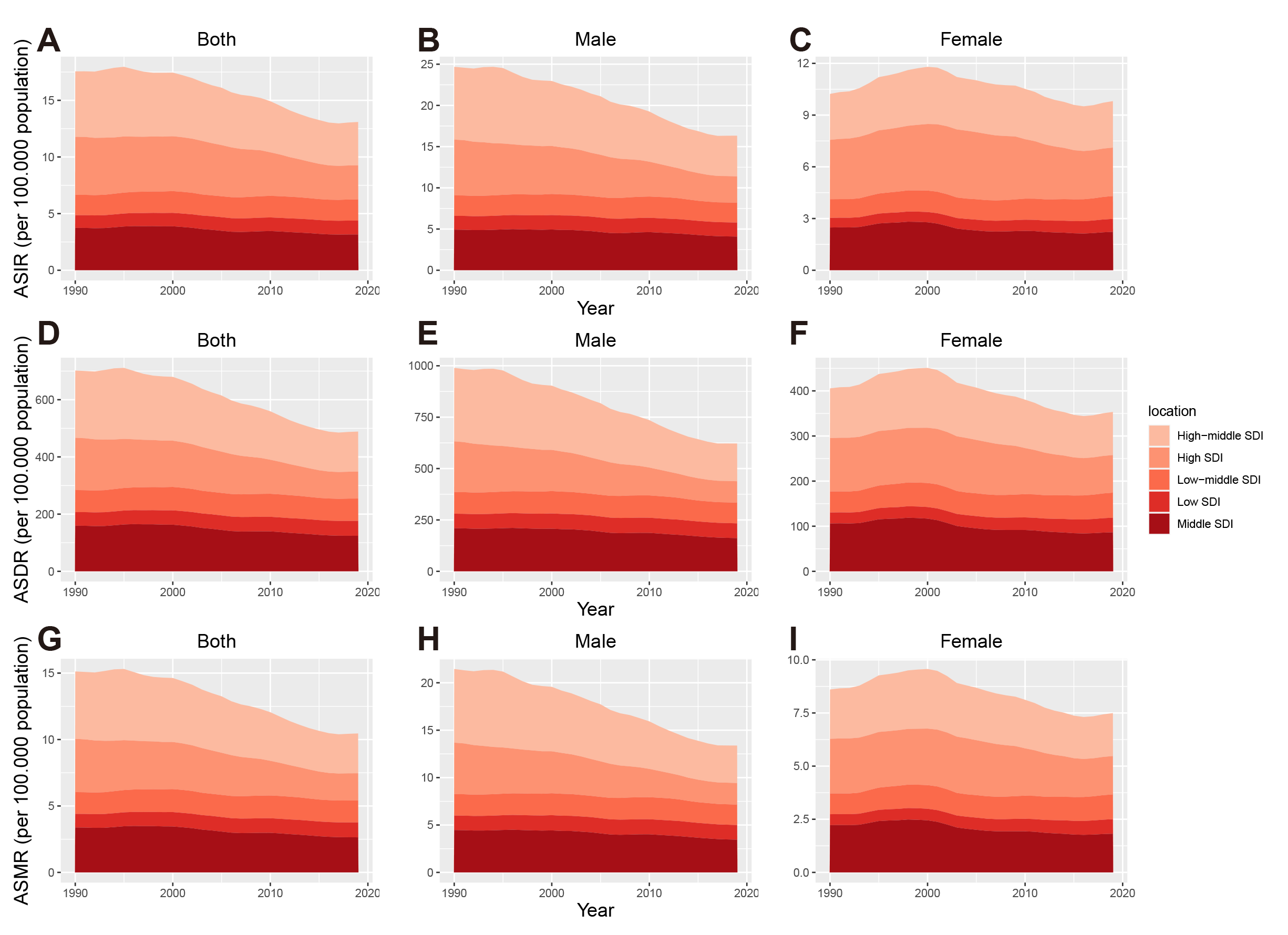

Supplement: Supplementary file 4 — Figure S1. The ASIR (A, B, C), ASDR (D, E, F), and ASMR (G, H, I) due to EO‐TBL cancer grouped by SDI quintiles for different sexes from 1990 to 2019. EO‐TBL cancer, early‐onset tracheal, bronchus, and lung cancer cancer; DALYs, disability‐adjusted life‐years; SDI, socio‐demographic index; ASIR, age‐standardized incidence rate; ASMR, age‐standardized mortality rate; ASDR, age‐standardized DALYs rate. [file TCA-15-601-s004.tif]

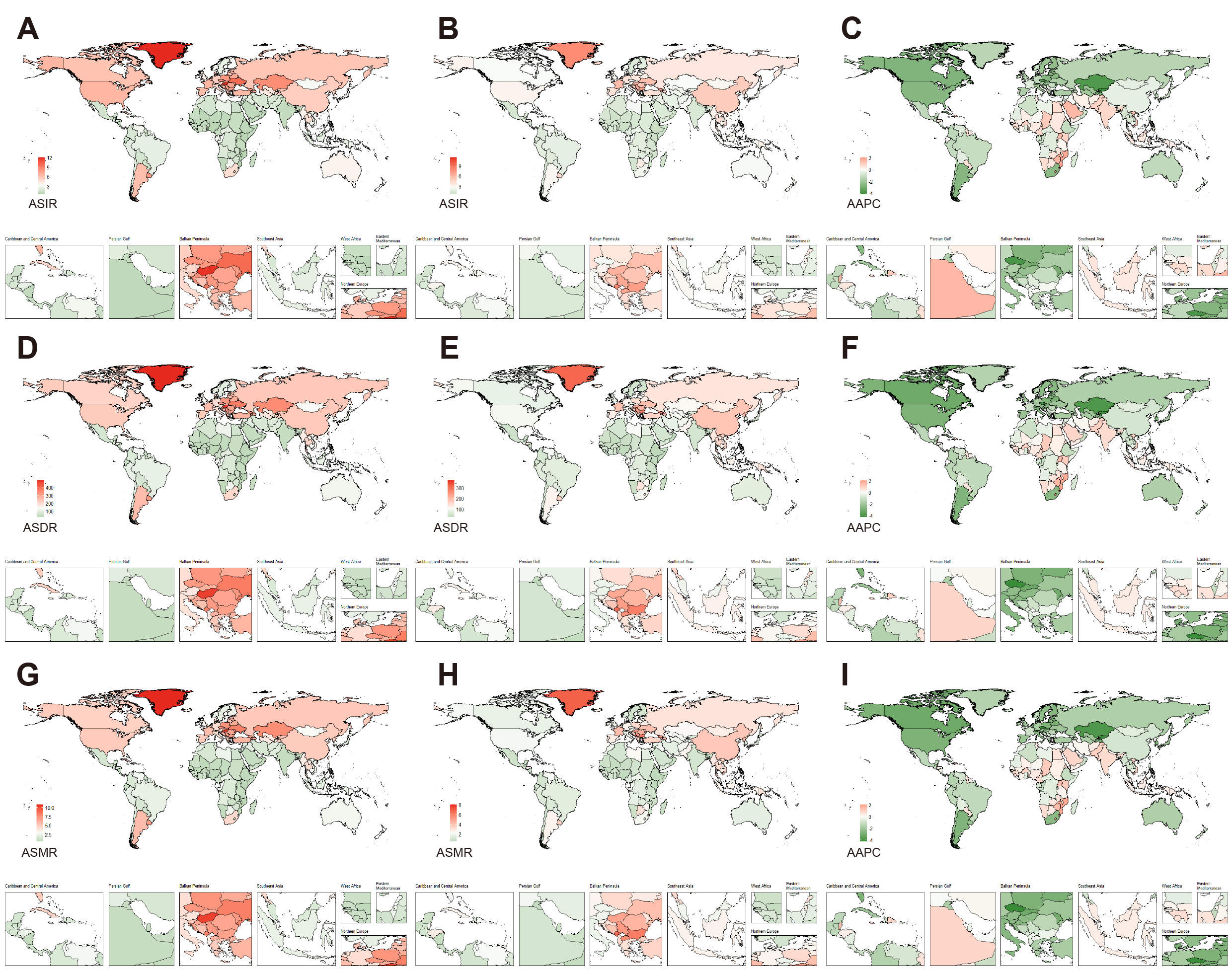

Supplement: Supplementary file 5 — Figure S2. Global maps of ASIR (A), ASDR (D), and ASMR (G) in 1990, global maps of ASIR (B), ASDR (E), and ASMR (H) in 2019, as well as AAPC in ASIR (C), ASDR (F), and ASMR (I) from 1990 to 2019. EO‐TBL cancer, early‐onset tracheal, bronchus, and lung cancer cancer; DALYs, disability‐adjusted life‐years; AAPC, average annual percent change; ASIR, age‐standardized incidence rate; ASMR, age‐standardized mortality rate; ASDR, age‐standardized DALYs rate. [file TCA-15-601-s002.tif]

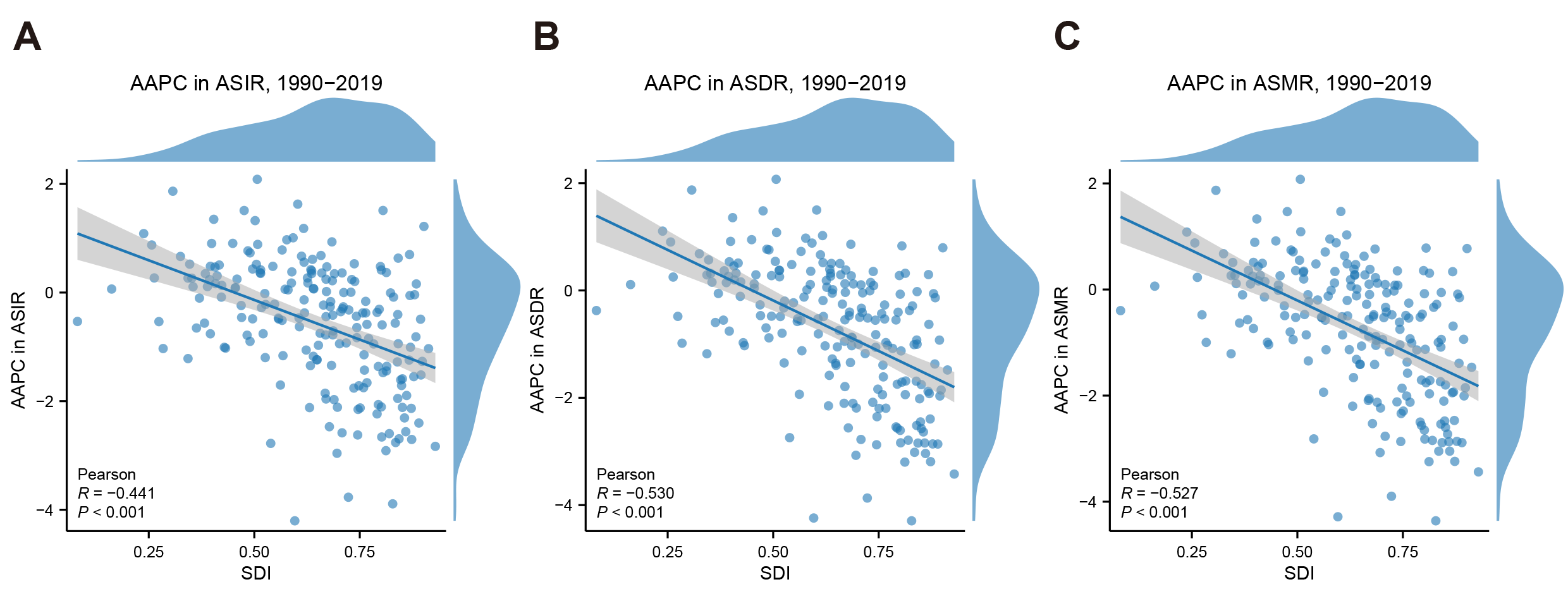

Supplement: Supplementary file 6 — Figure S3. AAPC of ASIR (A), ASDR (B), and ASMR (C) from 1990 to 2019 in 204 countries and territories according to the SDI in 2019. EO‐TBL cancer, early‐onset tracheal, bronchus, and lung cancer cancer; DALYs, disability‐adjusted life‐years; AAPC, average annual percent change; SDI, socio‐demographic index; ASIR, age‐standardized incidence rate; ASMR, age‐standardized mortality rate; ASDR, age‐standardized DALYs rate. [file TCA-15-601-s001.tif]
